# Joint synthesis of longitudinal MRI and enhanced prediction of neoadjuvant chemotherapy response in breast cancer: a multicohort study

**DOI:** 10.1186/s12885-026-15889-4

**Published:** 2026-03-24

**Authors:** Ming Fan, Songlin He, Chao Wan, Jiatong Zhang, Tao Tan, Shuyi Zhou, Shujun Chen, Chao You, Yajia Gu, Lihua Li

**Affiliations:** 1 Institute of Biomedical Engineering and Instrumentation, Hangzhou Dianzi University, Hangzhou, 310018 China; 2 Faculty of Applied Sciences, Macao Polytechnic University, Taipa Island, Macao, SAR 999078 China; 3 College of Arts and Human Sciences, University of Wisconsin-Stout, Menomonie, Wisconsin WI 54751 USA; 4 Zhejiang Cancer Hospital, Hangzhou Institute of Medicine (HIM), Chinese Academy of Sciences, Hangzhou, Zhejiang 310022 China; 5 Department of Radiology, Fudan University Shanghai Cancer Center, Shanghai, 200032 China

**Keywords:** Breast cancer, Generative adversarial network, Feature synthesis, Neoadjuvant Chemotherapy, Longitudinal MR Images

## Abstract

Neoadjuvant chemotherapy (NAC) induces critical morphological and vascular changes in breast cancer tumors, yet existing deep learning techniques in medical imaging tend to neglect these longitudinal changes throughout the treatment course. Here, we propose simultaneously synthesizing early-NAC MR images and improving the prediction of therapeutic responses from preoperative MRI. A cycle generative adversarial network (GAN)-based Longitudinal Image Synthesis and Prediction Convolutional Network (LISPCN) was developed by decoding preoperative MR images into a latent feature vector and then encoding it to jointly synthesize early-NAC DCE-MR images and predict therapeutic responses. This approach balances the shared information between the two tasks, thereby enhancing the overall model performance. We developed a specialized semantic and perceptual loss optimized for the multitask learning architecture, adopting an Efficient Channel Attention (ECA) module into the generators to enhance the model’s discriminative ability. Our LISPCN model surpassed others in image quality, achieving a structural similarity index of 0.909 and a peak signal-to-noise ratio of 34.995. For the internal validation dataset, the model achieved an AUC of 0.903, outperforming the model using solely preoperative image. For the external validation dataset, it achieved an AUC of 0.832. Model visualization demonstrates an increased focus on the lesion, indicating its capability to learn longitudinal changes during treatment. By leveraging intermediate NAC information, the LISPCN model enables the generation of early-stage NAC images from preoperative images, serving as a supportive tool to monitor treatment progress. This method has significant clinical implications for personalized diagnostic and treatment decisions in breast cancer patients.

**Supplementary Information:**

The online version contains supplementary material available at 10.1186/s12885-026-15889-4.

## Introduction

Neoadjuvant chemotherapy (NAC) is a standard treatment strategy for breast cancer management that aims to reduce tumor size, enhance surgical outcomes, and potentially enable breast-conserving surgery instead of mastectomy [1, 2]. Administration of NAC can yield significant benefits for a subset of patients, leading to the achievement of a pathological complete response (pCR) [3–6]. Nevertheless, some patients may suffer from poor response [7], which can be affected by side effects from treatment [8]. To optimize patient outcomes, ongoing research endeavors are focused on identifying predictive factors that can help identify patients who are more likely to achieve pCR following NAC.

Throughout the 6–8 cycles of NAC for breast cancer, considerable morphological and functional changes in tumor characteristics occur [9]. Specifically, concentric shrinkage during NAC has been correlated with improved patient survival. Furthermore, elevated residual cancer burdens have been identified as predictors of less favorable clinical outcomes after receiving NAC [10, 11]. Therefore, monitoring factors that impact the tumor response during NAC can provide crucial prognostic insights, inform therapeutic decisions and predict patient outcomes.

Dynamic contrast-enhanced magnetic resonance imaging (DCE-MRI) serves as a noninvasive approach for assessing breast tumors by capturing kinetic patterns, enabling the assessment of tumor architecture, vasculature, and perfusion characteristics for NAC response prediction [12–14]. DCE-MRI features were generated from other parametric images such as T2 weighted imaging using a generative adversarial model for a better tumor diagnosis [15]. Preoperative DCE-MRI was used for predicting the response to NAC by leveraging both machine learning-based [16–19] and deep learning-based approaches [20]. A previous study revealed that reduced tumor heterogeneity, as indicated by texture features, was associated with pCR after NAC in breast cancer patients [21]. Furthermore, radiomic features extracted from early-stage NAC images have demonstrated superior predictive performance compared to those derived solely from preoperative images [22]. Recent advancements have involved the integration of longitudinal MRI features from both the preoperative and intermediate treatment phases to predict NAC outcomes more effectively [23]. Nonetheless, the prevalent approach frequently entails training computationally demanding models that convert imaging data into radiomics or deep radiomics, which are subsequently reduced into dichotomized clinical outcome indicators. This necessitates an extensive parameter space for model training to optimize the predictive performance. Therefore, these data-driven studies exclusively emphasize eventual treatment outcomes, disregarding morphological or functional alterations that occur throughout the course of treatment, particularly in the early phase.

Our study diverges from traditional practices, which typically involve the utilization of preoperative or longitudinal imaging to train predictive models linked to dichotomized NAC outcomes. Instead, we developed a deep multitask convolutional neural network (CNN) that integrates a generative modeling framework informed by the longitudinal alterations of tumors during NAC. This advanced CNN is engineered to simultaneously predict NAC response and synthesize DCE-MR images during the early phase of NAC treatment. Our methodology holds the potential to improve clinical decision-making processes and yield critical insights into the temporal evolution of tumors in the early treatment stages.

The main contributions are summarized as follows, highlighting several notable strengths compared to previous investigations: First, we developed a multitask deep GAN model that effectively facilitates the prediction of pathological response to NAC and the generation of early NAC images. This model enables a comprehensive assessment of treatment response and visualization of early tumor changes, addressing both prediction and image generation tasks simultaneously. Combining GAN models with classification models significantly improved the predictive performance in classifying NAC responses based solely on preoperative images, as it facilitates in-depth learning of treatment-related changes, culminating in more precise outcome prediction. Second, we introduced a latent deep feature mapping technique that leverages preoperative DCE-MRI data. By integrating this latent feature, we strike a balance between the information contained in early NAC images and treatment outcomes. This integration enhances the accuracy and robustness of our predictions, as it captures the underlying characteristics of the tumor during treatment. Third, our framework incorporates constraints on both baseline and early treatment image generation. These constraints ensure that the generated images accurately reflect the observed changes in the tumor during NAC treatment. By including these constraints, we enhance the reliability of our predictions and provide a more comprehensive understanding of how the tumor evolves and the eventual treatment outcome.

## Materials and methods

### Pipeline illustration

**Fig. 1:**
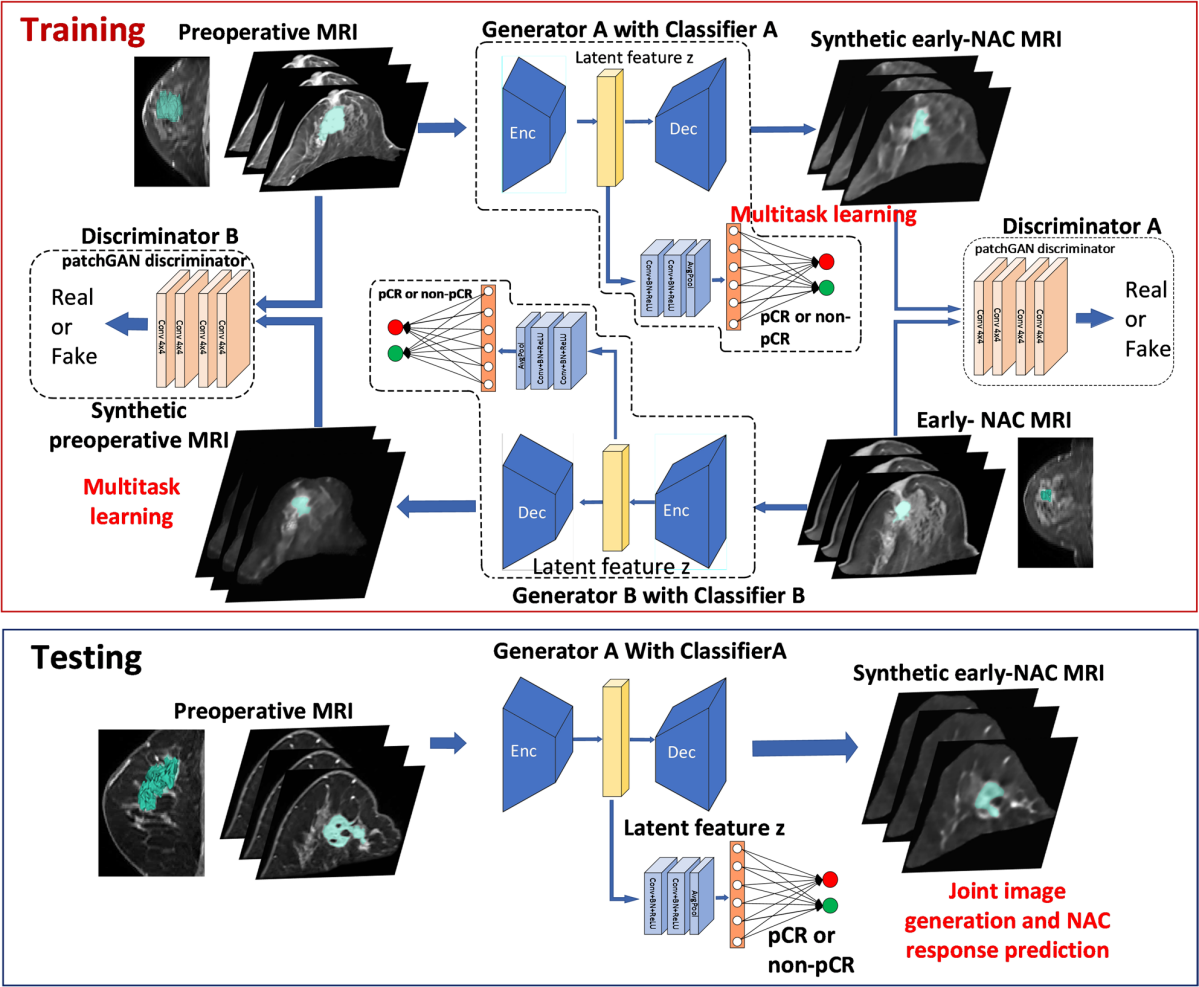
Framework overview. The Longitudinal Image Synthesis and Prediction Convolutional Network (LISPCN) is a framework specifically designed to generate images of early chemotherapy and predict response to neoadjuvant chemotherapy (NAC). It utilizes a cycle generative adversarial network (cycle GAN) to transform preoperative MRI images into their early-NAC phase counterparts for enhanced feature extraction and performance. After training on a development dataset, LISPCN was validated both internally and externally, demonstrating its ability to generate synthetic post-chemotherapy MRI images and accurately predict treatment responses

This study introduces the longitudinal image synthesis and prediction convolutional network (LISPCN), a tailored framework for generating synthetic early chemotherapy images and forecasting NAC outcomes (Fig. 1). Initially, discriminative features are extracted from preoperative and early treatment MRI scans via an encoder module. Utilizing a cycle generative adversarial network (cycle GAN) approach [24], the model transforms preoperative images into their early-NAC counterparts and vice versa (Supplementary Fig. S1). The encoder produces a latent feature map *z*, which is decoded to jointly yield synthetic early-NAC MRIs and predict treatment responses. Leveraging transfer learning and a perceptual loss strategy, the feature generation process is refined for enhanced performance; features are extracted using an ImageNet-trained VGG and ResNet architecture (Supplementary Fig. S2). The LISPCN, trained on a development dataset, was then employed on an internal validation cohort, taking preoperative MR images as input. To achieve this, the encoder module generates a latent feature map z, which is subsequently decoded to produce synthetic post-chemotherapy MRI and predict the response to treatment. This model was further validated in an independent external dataset to evaluate image generation and NAC response prediction.

### Dataset

The multicohort study included breast cancer patients, as illustrated in Table 1. This study was approved by the Institutional Review Board (IRB) from Fudan University Shanghai Cancer Center (2003214-7). Given its retrospective design, informed consent was waived by the Ethics Committee of Fudan University Shanghai Cancer Center. For the development and internal validation datasets, women who underwent NAC and had pre- and early NAC images available were enrolled (between March 2017 and November 2019). The external validation dataset was obtained from the I-SPY 2 trial, with a subset of 154 cases previously documented in references [25, 26].

**Table 1:** Patient Information

Characteristic	Development (*n* = 187)	Internal validation (*n* = 61)	External validation (*n* = 189)
Age	49.00 26–67	49.10 27–65	48.68 25–71
Ki-67			
High	108 (57.8)	38 (62.3)	-
Low	59 (31.6)	17 (27.9)	-
N/A	20 (10.6)	6 (9.8)	-
HER2			
Positive	113 (60.4)	34 (55.7)	46 (24.3)
Negative	55 (29.4)	21 (34.5)	143 (75.7)
N/A	19 (10.2)	6 (9.8)	0
ER			
Positive	98 (52.4)	28 (45.9)	-
Negative	71 (38.0)	27 (44.3)	-
N/A	18 (9.6)	6 (9.8)	-
pCR			
Response	34 (18.2)	12 (19.7)	70 (37.0)
No Response	153 (81.8)	49 (80.3)	119 (63.0)

**Fig. 2:**
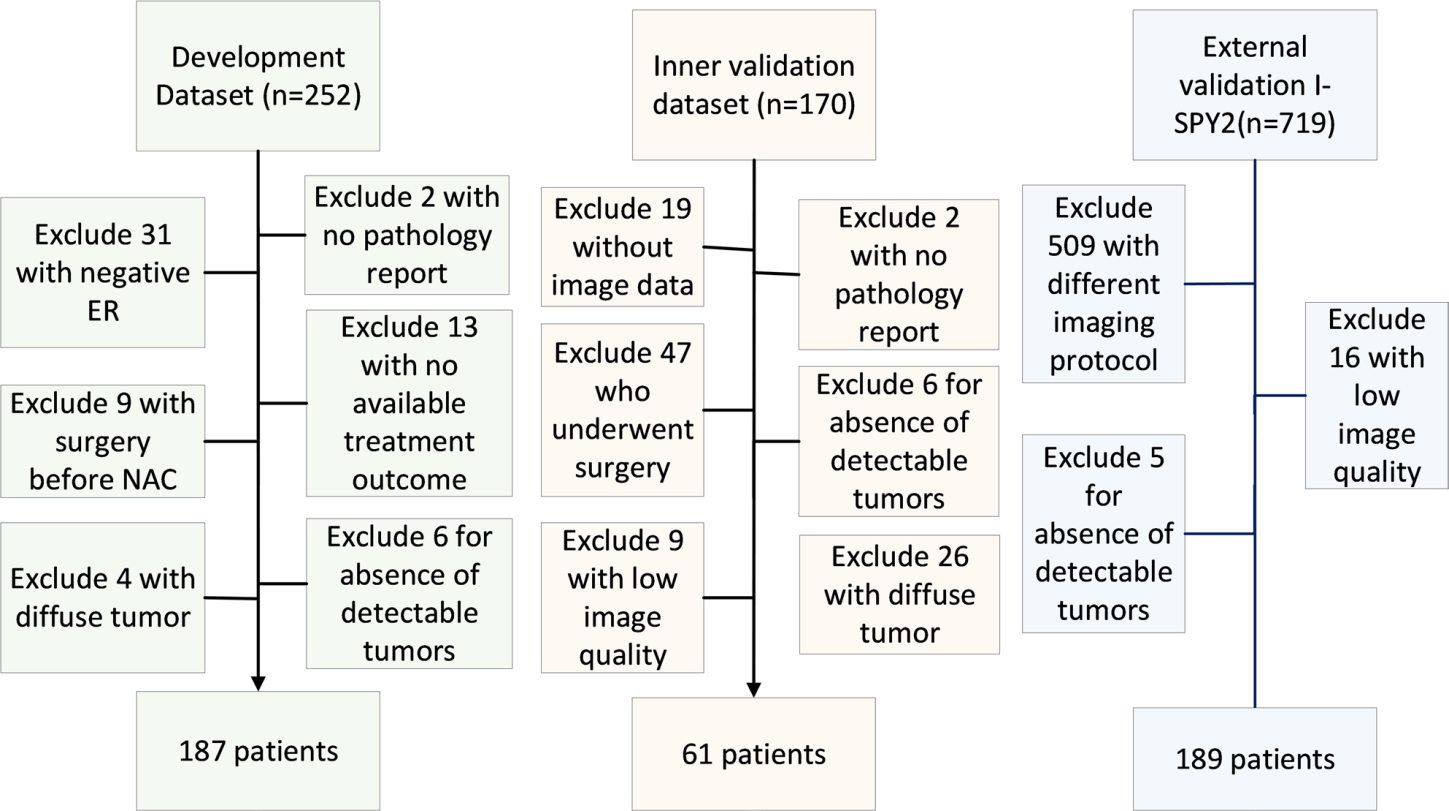
Data collection flow chart

The study’s inclusion criteria were as follows: First, all patients underwent 6 or 8 cycles of NAC using taxotere–epirubicin–cyclophosphamide (TEC) [27]. Second, the participants did not receive any surgical or other treatments, including chemotherapy. Third, MRI scans before and after the first cycle of NAC were available. The same imaging protocol was used for the acquired image scans at both the first and second chemotherapy cycles. Fourth, clinicopathological response information was needed. The data collection flowchart is illustrated in Fig. 2.

The three datasets consisted of 437 women for development and inner and external validation of the proposed framework. The samples in the development dataset had a mean age of 49.00 years (range, 27–65 years), while they were 49.10 years (range, 27–65) and 48.68 years (range, 25–71) in the inner and external validation datasets, respectively. For the three datasets, 34 (18.2%), 12 (19.6%) and 70 (37.0%) patients achieved pCR. We confirmed that patient-level separation was maintained in all dataset splits.

### Image preprocessing and imaging protocols

To optimize the computational efficiency of the deep learning model, extraneous anatomic structures, such as the skin and chest wall, which are not pertinent to the breast area, were excised utilizing CPFNet [28]. The efficacy of segmentation was quantitatively assessed by determining the pixelwise disparity between the predicted semantic segmentation output and the reference mask annotations. The concordance between the segmented output and the ground truth masks was gauged by employing the Dice similarity coefficient. Subsequently, the DCE-MR images, devoid of nonrelevant tissues, were processed as inputs to the CNN. The network was exclusively trained on unilateral breast regions exhibiting solid neoplastic lesions.

Imaging data collected from the multicohort datasets. For the development and internal validation dataset, MR images were obtained using a 3.0 T T1-weighted system (Siemens Magnetom Verio, Erlangen, Germany) equipped with an eight-channel bilateral breast coil and employing fat suppression. Precontrast images were acquired prior to contrast agent administration. Subsequently, a dose of 0.1 mmol/kg gadobutrol was intravenously injected at a rate of 2 mL/second. DCE-MRI acquisition involved a precontrast (S0) sequence, followed by five postcontrast series (S1 to S5) with a temporal resolution of 83–43 s. The detailed imaging parameters are listed in Table 2. All MRI scans from the external validation dataset were conducted using DCE-MRI following the preestablished I-SPY 2 MRI guidelines.

**Table 2:** Imaging parameters

Parameters	Dataset 1	Dataset 2	Dataset 3
Magnetic Field Strength	3.0T	3.0T	1.5T/3T
Time of Repetition, TR	4.51ms	4.5ms	4.1-5.23ms
Time of Echo, TE	1.61ms	1.56ms	1.33-2.42ms
Flip Angle, FA	10°	90°	10/12/15/17.7/19
Field of View, FOV	340 × 340 mm	360 × 360	300–380 mm
Acquisition Matrix	896 × 896	384 × 384	256/384/416/512
Slice Thickness	2.4 mm	2.2 mm	0.9–2.5 mm
Pixel Spacing	0.379 mm	0.938 mm	0.59–1.4 mm
Slice Number	72	80	64/72/80/88/96/112/144/160/176/208
Enhanced Series	1 + 5	1 + 8	4/5/6/7/8/9
Enhancing Interval	83s	43s	60–122 s

### Histological analysis

Tumor histology and receptor status were established via immunohistochemistry (IHC) on core biopsies. Hormone receptor (HR) positivity was defined as ≥ 1% nuclear staining for estrogen receptor (ER) or progesterone receptor (PR). human epidermal growth factor receptor 2 (HER2) status was categorized as negative with IHC 0 or 1+, positive with IHC 3+, and equivocal cases (IHC 2+) were further evaluated using fluorescence in situ hybridization (FISH). Ki-67 < 14% was considered low expression. Pathological assessment of surgical specimens determined pCR, with a Miller–Payne score of 5 indicating pCR and scores < 5 classified as non-pCR [29].

### Network structure

A cycle GAN [24] served as the primary architecture for synthesizing early-NAC images, as delineated in Supplementary Fig. S1. This dual-branch framework incorporates paired generators (denoted as *G* and *F*) alongside corresponding adversarial discriminators (identified as DEC_1_ and DEC_2_) to facilitate the transformation and evaluation of image authenticity. Each generator employs an encoder composed of a succession of three convolutional layers featuring an ascending channel configuration of 2, 4, and 8 to effectively extract features (Supplementary Fig. S2). We have integrated an efficient channel attention (ECA) module [30] within the generators to amplify the model’s discriminative capacity, enabling a more targeted emphasis on pivotal image regions and thereby bolstering both the classification and generative performance of the model. The discriminator component of the architecture is grounded in the Patch-GAN framework [31], a design renowned for its capacity to promote a more stable training process and to improve the quality of the synthesized images.

Our study proposes a generator coupled with a classifier to preserve the discriminatory information for NAC response prediction inherent in the mapped latent feature *z*. As illustrated in Supplementary Fig. S1, the classifier and decoder components concurrently leverage the latent feature representation *z* generated by the encoder to execute both predictive and generative functions. This approach equilibrates the shared information between the two, thereby enhancing the overall model performance. Detailed loss functions are provided in Supplementary Methods.

### Training strategy

For DCE-MRI, the image series (S0, S3 and S5) were included as a three-channel input. During the training of the GAN, paired pre-NAC and early-NAC data from the same patient were used as inputs to the network. Data augmentation techniques were applied to the existing data in this study on the patient image slice samples in the training set. The augmentation techniques included horizontal mirroring, vertical mirroring, and rotation at specified angles [90°, 180°, 270°].

The encoder, decoder, generator, and discriminator were trained using the Adam optimizer with hyperparameters $\:{\beta\:}_{1}=0.5$ and $\:{\beta\:}_{2}=0.99$ and a batch size of 8. The initial learning rates for generative adversarial and classification were set to 2e-3 and 1e-4, respectively. The learning rate was decreased by multiplying by 0.97 every 50 epochs.

The proposed framework was implemented on a workstation equipped with two NVIDIA Quadro RTX6000 GPUs. The total training time was approximately 15 h. The training and testing durations for each epoch were 2.702 s and 0.412 s, respectively.

### Ablation experiments

We performed four ablation experiments to assess our model’s predictive efficacy (Supplementary Fig. S3). For each experiment, we excluded one component from the full network architecture and retrained the model to demonstrate the contribution of that specific element to the model’s performance. The exclusion criteria were as follows: 1) Decoder: The model involves omitting the decoder component of the model (denoted AS-Dec). By doing so, the architecture is simplified to a conventional CNN without generative capabilities. 2) Discriminator: The discriminator is removed, resulting in a hybrid structure that combines an autoencoder with a classification module (denoted AS-Dis). This evaluates the discriminator’s role in ensuring synthetic image realism and feature alignment. 3) Generator: By removing the generator from the architecture, the cycle consistency loss is no longer applicable, reducing the architecture to a configuration akin to the Pix2Pix model, as described in [31] (denoted AS-GF). 4) Perceptual: The elimination of this component aims to quantify its contribution to the model’s feature extraction capability (denoted AS-Pcp).

### Statistical analysis

The proposed model was trained on the development dataset using preoperative MR images, validated on an internal cohort, and subsequently tested on the independent ISPY2 external dataset for image generation and NAC response prediction. Given the differences in imaging parameters and histological characteristics between the external validation cohort and the datasets used for model development and internal validation, we fine-tuned the model using a portion (20%) of the samples from the external validation cohort. Subsequently, the fine-tuned model was applied to the remaining samples to assess its predictive performance. The model had been fine-tuned for 20 epochs with a reduced learning rate of 5 × 10⁻⁶, where only the generator’s classification head weights were updated using both preoperative MRI and synthesized early-NAC imaging data. To evaluate the performance in predicting the response to NAC, we calculated the accuracy, precision, sensitivity, specificity, and area under the curve (AUC) values.

DenseNet-169 [32] was adopted as the backbone for the feature extractor and was compared with the U-Net [33], ResNet-50 [34], EfficientNet [35] and MobileNet [36] models for NAC response prediction. The ECA attention mechanism [30] was employed to optimize the ResNet and MobileNetV2 models. We also compared the proposed LISPCN with other image generation methods, including pix2pix [31] and CycleGAN [24].

The feature-level quality was evaluated by the Fréchet inception distance (FID) [37] and the learned perceptual image patch similarity (LPIPS) value by measuring the similarity of the mapped features and the real MRI features [38]. The image-level quality was assessed by structural similarity (SSIM) and the peak signal-to-noise ratio (PSNR). To assess the response of GAN-based and other imaging-based models to NAC, we utilized a class activation map (grad-CAM plus)-based model [39, 40]. We used the intersection over union (IoU) metric to quantitatively evaluate the attention activation areas of the LISPCN model and CNN models.

## Results

### Generation quality assessment of the synthesized early-NAC image

**Fig. 3:**
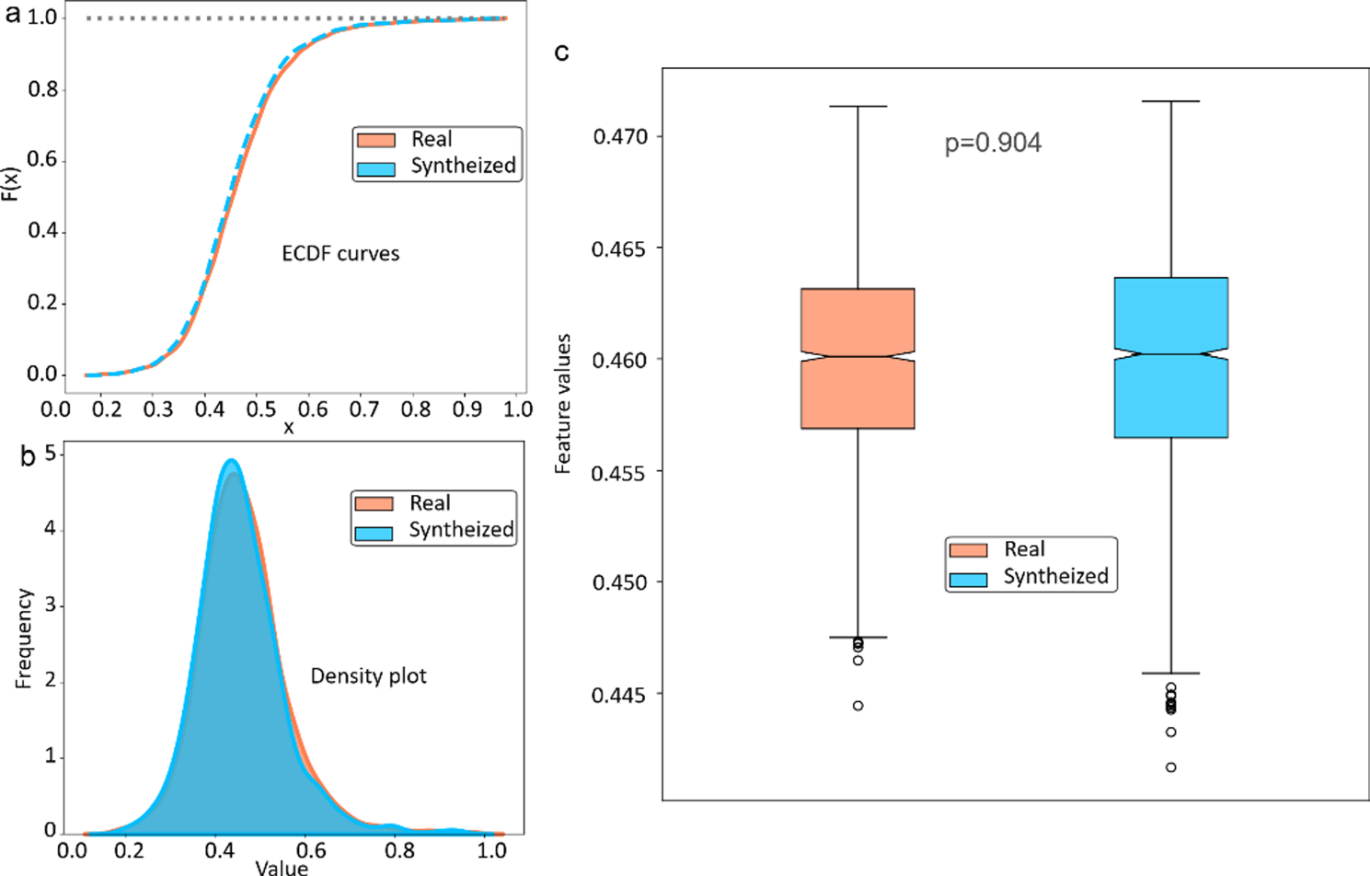
Feature distribution of the real and synthesized early-NAC MRI features. **A** The empirical cumulative density of the real and synthetic image features. **B** Density plot of the real and generated image features. **C** Boxplot of the real and synthetic image features showing no significant difference

Figure 3 demonstrates a significant overlap in the probability density distributions of features between the synthesized and real early-NAC images, suggesting the model’s effectiveness in learning the intrinsic characteristics of real early-NAC images and the generation of high-quality images. The similarity in the empirical distribution functions, as shown in Fig. 3b, further supports the minimal practical differences between the two sets of features. Statistical analysis confirmed that there was no significant difference in the features between the synthesized and real early NAC images (*p* = 0.904).

### Comparison of various methods for image generation

**Fig. 4:**
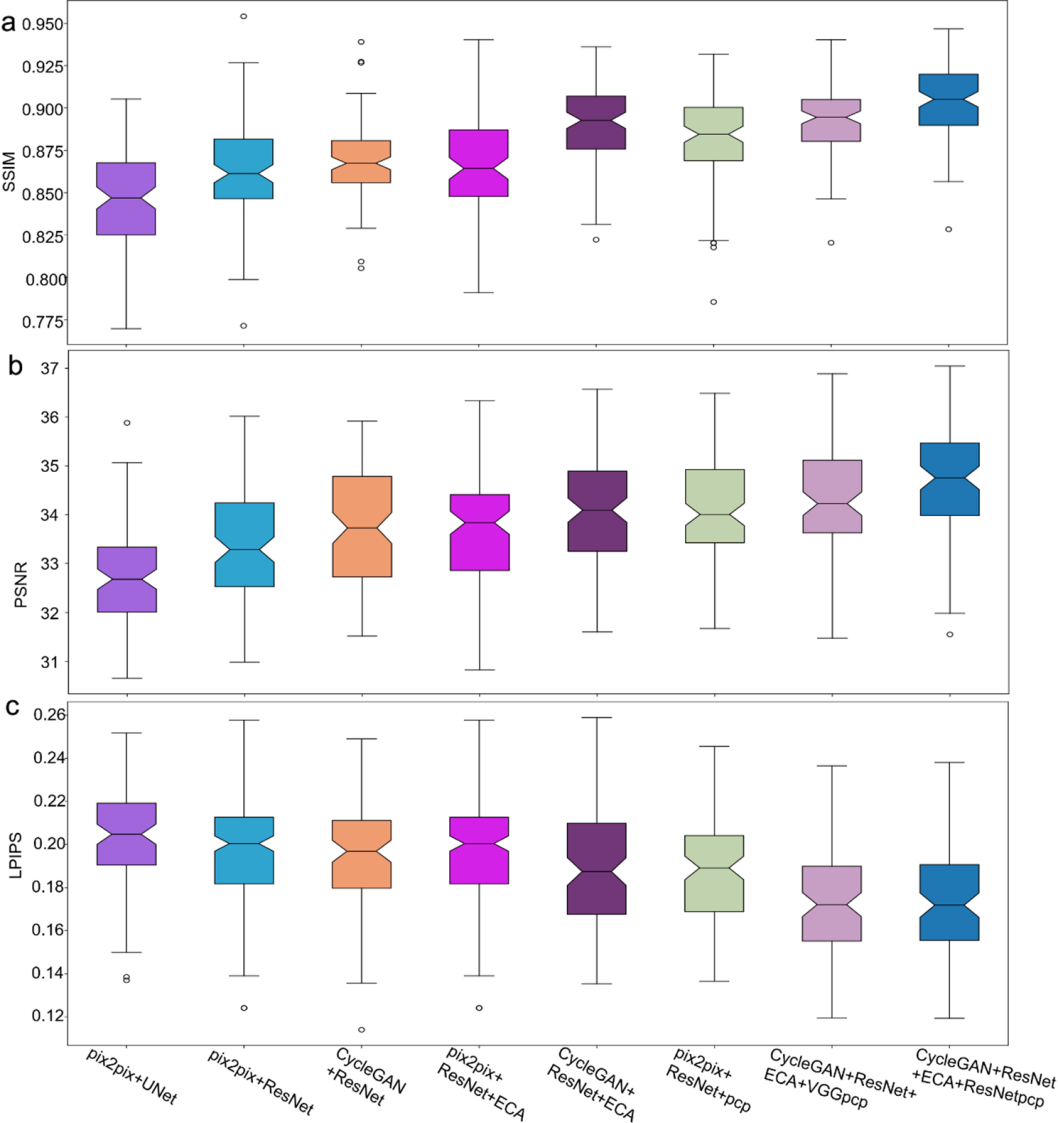
Comparison of different models in terms of **a**) SSIM **b**) PSNR and **C**) LPIPS

Our comparative analysis of various techniques for feature-level and image-level synthesis revealed superior performance when employing CycleGAN in conjunction with a ResNet-based feature extractor enhanced with ECA and perceptual loss modules (Table 3 and Fig. 4). The CycleGAN framework for image generation consistently outperformed Pix2Pix in terms of image-level and feature-level evaluation metrics. Moreover, the incorporation of ECA and perceptual loss, leveraging a pretrained ResNet model, significantly improved feature representation capabilities compared to configurations omitting these enhancements.

**Table 3:** Performance of image generation methods using different architectures

Generator model	SSIM (95%CI)	PSNR (95%CI)	LPIPS (95%CI)	FID
Pix2Pix + U-Net	0.846 ± 0.054	32.80 ± 1.98	0.213 ± 0.037	33.65
Pix2Pix+ResNet	0.863 ± 0.057	33.49 ± 2.01	0.195 ± 0.055	31.74
CycleGAN+ResNet	0.868 ± 0.032	33.76 ± 1.74	0.194 ± 0.052	25.17
Pix2Pix+ResNet + ECA	0.867 ± 0.058	33.69 ± 2.32	0.196 ± 0.054	30.34
CycleGAN+ResNet + ECA	0.883 ± 0.042	33.98 ± 2.17	0.188 ± 0.061	23.61
Pix2Pix+ResNet + ECA+pcp^1^	0.879 ± 0.044	34.11 ± 2.04	0.187 ± 0.048	27.89
CycleGAN+ResNet + ECA+pcp^2^	0.890 ± 0.040	34.19 ± 2.20	0.174 ± 0.051	22.27
CycleGAN+ResNet + ECA +pcp^1^	0.901 ± 0.038	34.56 ± 1.94	0.174 ± 0.052	19.83

^1^The perception pretrained from imageNet with ResNet structure

^2^The perception pretrained from ImageNet with VGG structure

### Evaluation of the performance of the LISPCN in NAC response prediction

The efficacy of the proposed methodology for NAC response prediction using preoperative MR images was evaluated in the internal validation dataset. The comparative analyses included prevalent CNN architectures utilizing preoperative images without GAN integration (Supplementary Fig. S4). The findings demonstrated that the proposed method achieved superior predictive accuracy, with an AUC of 0.903 on the internal validation dataset. This performance was significantly higher than that of CNN-based models (ResNet50, DenseNet169, MobileNetV2, ShuffleNetV2, EfficientNet) as well as Transformer-based models, including the vision transformer (ViT) [41], Swin-Transformer [42], and hybrid CNN-Transformer (Table 4).

**Table 4:** Comparison of predictive models with different network backbones

Model	AUC (95%CI)	Accuracy	Precision	Sensitivity	Specificity	F1	*p* value
ResNet50 ^†^	0.680 ± 0.063	0.664	0.683	0.489	0.782	0.570	0.00011
ResNet50 ^‡^	0.836 ± 0.026	0.776	0.846	0.758	0.801	0.800	0.0048
DenseNet169 ^†^	0.650 ± 0.057	0.637	0.692	0.635	0.663	0.662	7.77e-05
DenseNet169 ^‡^	0.848 ± 0.026	0.784	0.844	0.778	0.793	0.810	0.0059
MobileNetV2 ^†^	0.660 ± 0.081	0.657	0.698	0.643	0.629	0.669	1.13e-05
MobileNetV2 ^‡^	0.820 ± 0.029	0.745	0.800	0.757	0.729	0.777	0.0137
ShuffleNetV2 ^†^	0.688 ± 0.097	0.638	0.649	0.580	0.695	0.613	1.53e-05
ShuffleNetV2 ^‡^	0.827 ± 0.029	0.755	0.816	0.754	0.756	0.784	0.0372
EfficientNetV2 ^†^	0.707 ± 0.073	0.639	0.760	0.562	0.749	0.646	2.48e-06
EfficientNetV2 ^‡^	0.834 ± 0.031	0.757	0.829	0.739	0.782	0.782	0.0032
VIT ^†^	0.784 ± 0.023	0.706	0.886	0.576	0.893	0.698	1.55e-11
VIT ^‡^	0.883 ± 0.022	0.785	0.790	0.867	0.673	0.825	0.0239
SwinT ^†^	0.766 ± 0.028	0.678	0.770	0.648	0.720	0.704	0.003
SwinT ^‡^	0.862 ± 0.018	0.778	0.819	0.802	0.742	0.810	5.48e-06
CNN-Transformer ^†^	0.773 ± 0.018	0.718	0.696	0.924	0.422	0.794	4.13e-05
CNN-Transformer ^‡^	0.871 ± 0.014	0.798	0.941	0.703	0.936	0.805	3.06e-04
LISPCN	0.903 ± 0.021	0.813	0.835	0.848	0.763	0.842	-

^†^ Model trained and independently tested using pre-NAC images

^‡^ Model trained and independently tested using early-NAC images

We also conducted comparative experiments with these models using early-NAC images (Table 4). The experimental results revealed that although these baseline models exhibited improved performance with early-NAC images compared to pre-NAC images, they consistently underperformed relative to our LISPCN framework. This finding suggests that our model benefits from effectively integrating information from both imaging phases (pre- and early-NAC) through its generative model component.

**Fig. 5:**
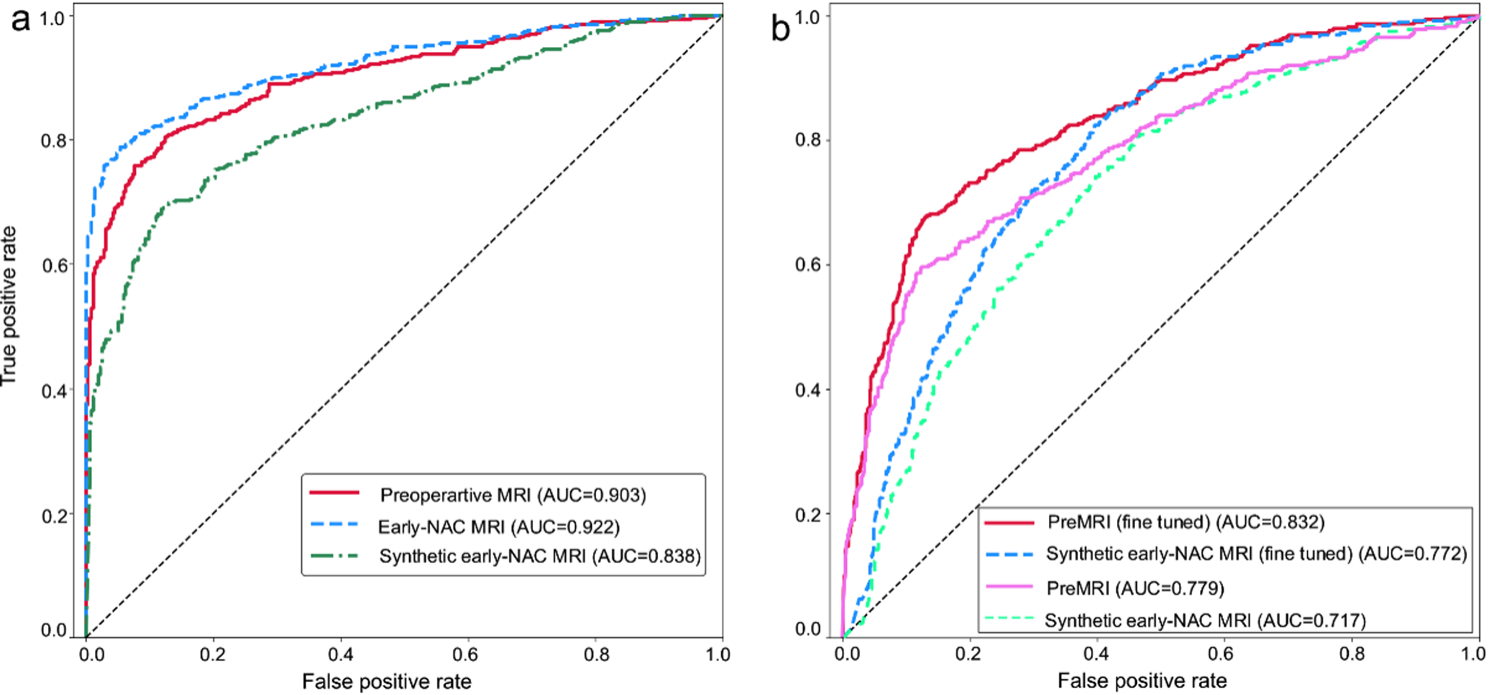
Validation of the proposed model for pCR prediction. **A** The predictive model in the internal validation dataset utilizing preoperative, synthesized early-NAC and real early-NAC data. **B** The predictive model in the external validation cohort utilizing preoperative MRI and synthesized early-NAC MRI. The models were evaluated after fine-tuning

The performance of the LISPCN was also evaluated using preoperative, synthesized early-NAC and real early-NAC data in the inner and external validation datasets (Fig. 5 and Table 5). Within the internal validation set, the model yielded an AUC of 0.838 for latent features using synthesized early-NAC MRI and 0.922 for features generated from real early-NAC MRI. In the external validation cohort, the latent feature derived from preoperative MRI initially yielded an AUC of 0.779, based exclusively on the testing samples from the I-SPY2 dataset. Subsequently, after fine-tuning the model using both preoperative MRI and synthesized early-NAC images, this AUC improved to 0.832. Notably, the latent feature from synthesized early-NAC MRI shows an initial AUC of 0.717, which increased to 0.772 following fine-tuning.

**Table 5:** Evaluation of the LISPCN using preoperative, early-NAC and synthesized early-NAC images

	Input Image	AUC (95%CI)	Accuracy	Sensitivity	Specificity	F1
Internal validation dataset	Preoperative	0.903 ± 0.021	0.813	0.848	0.763	0.842
	Early-NAC	0.922 ± 0.020	0.848	0.830	0.873	0.865
	Synthetic	0.838 ± 0.029	0.763	0.796	0.715	0.797
Extensional Validation	Preoperative	0.779 ± 0.038	0.715	0.681	0.736	0.643
	Preoperative*	0.832 ± 0.033	0.749	0.772	0.735	0.699
	Synthetic	0.717 ± 0.035	0.611	0.844	0.470	0.620
	Synthetic*	0.772 ± 0.038	0.637	0.914	0.469	0.656

*fine-tuned model

### Visualization of the model in NAC response prediction

**Fig. 6:**
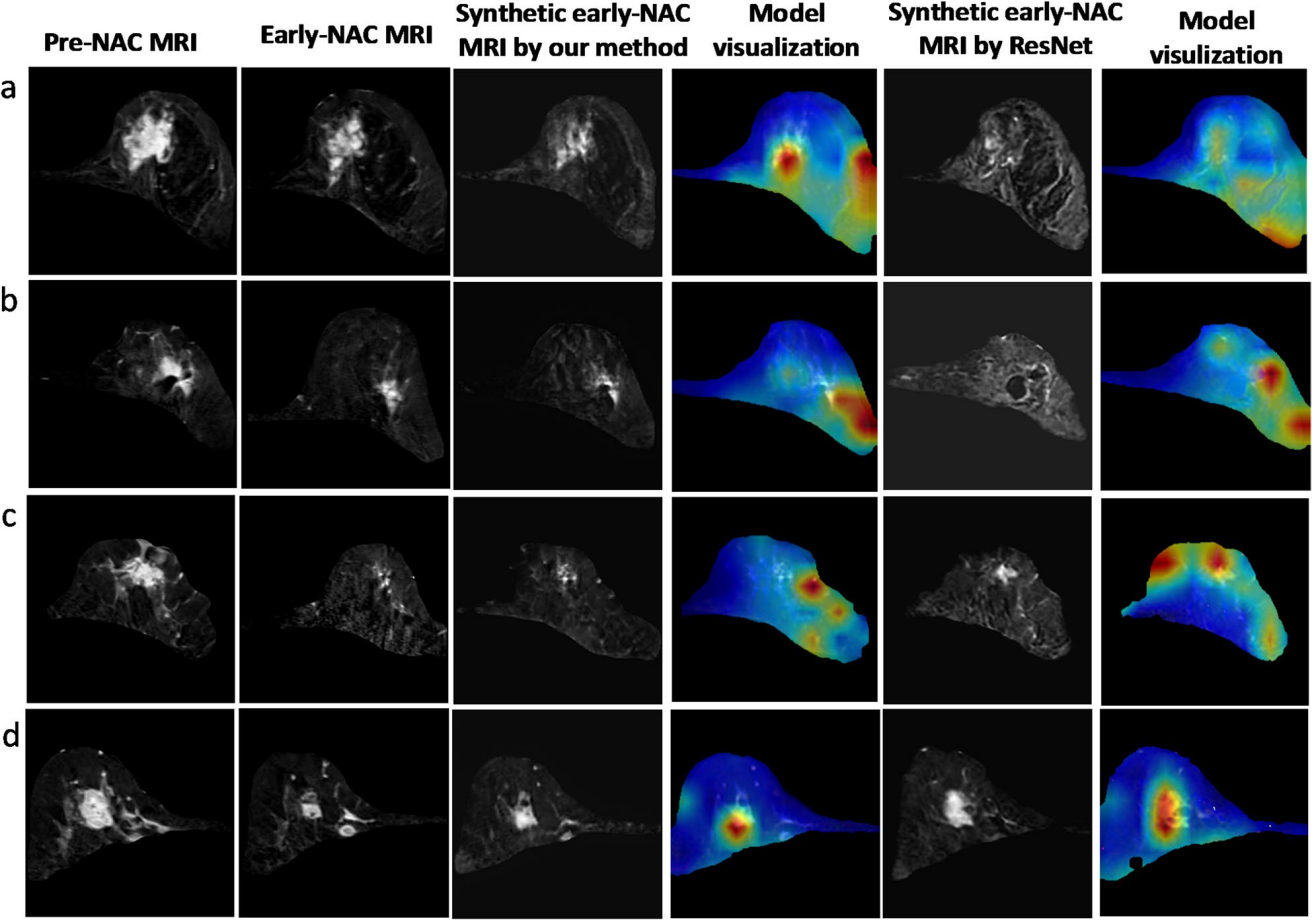
Model visualization examples for joint image generation and neoadjuvant chemotherapy (NAC) response prediction. From left to right, the columns display the preoperative MRI, the real early-NAC MRI, the synthetic early-NAC MRI images generated by the proposed method, the visualization of the proposed model, the synthetic early-NAC images created by a standard GAN model, and the corresponding class activation map for comparison. **A** A 50-year-old female patient exhibiting a non-pathological complete response (non-pCR) to NAC. **B** A 40-year-old female patient demonstrating a non-pCR following NAC. **C** A 61-year-old female patient who achieved a pCR. **D** A 49-year-old female patient who also achieved a pCR subsequent to NAC treatment

Figure 6 shows the results of comparative visualization of the predictive models for NAC outcome prediction. The LISPCN model’s attention maps show focused activations within the lesion, indicating lesion-centric predictions. In contrast, the CNN model’s attention maps exhibit more diffuse activation patterns. Through adversarial training, the LISPCN model precisely identifies lesion features and integrates early NAC imaging characteristics, which is crucial for assessing treatment response. These findings demonstrated that heightened attention within the lesion was correlated with a higher pCR prediction probability. Conversely, the common CNN model directs more attention to nonlesion regions, reducing prediction accuracy and resulting in potential misclassification of the therapeutic response. Additionally, all the examples showed that our model’s attention maps align more closely with the lesion areas than do those from the CNN model, suggesting superior performance in therapeutic prediction.

**Fig. 7:**
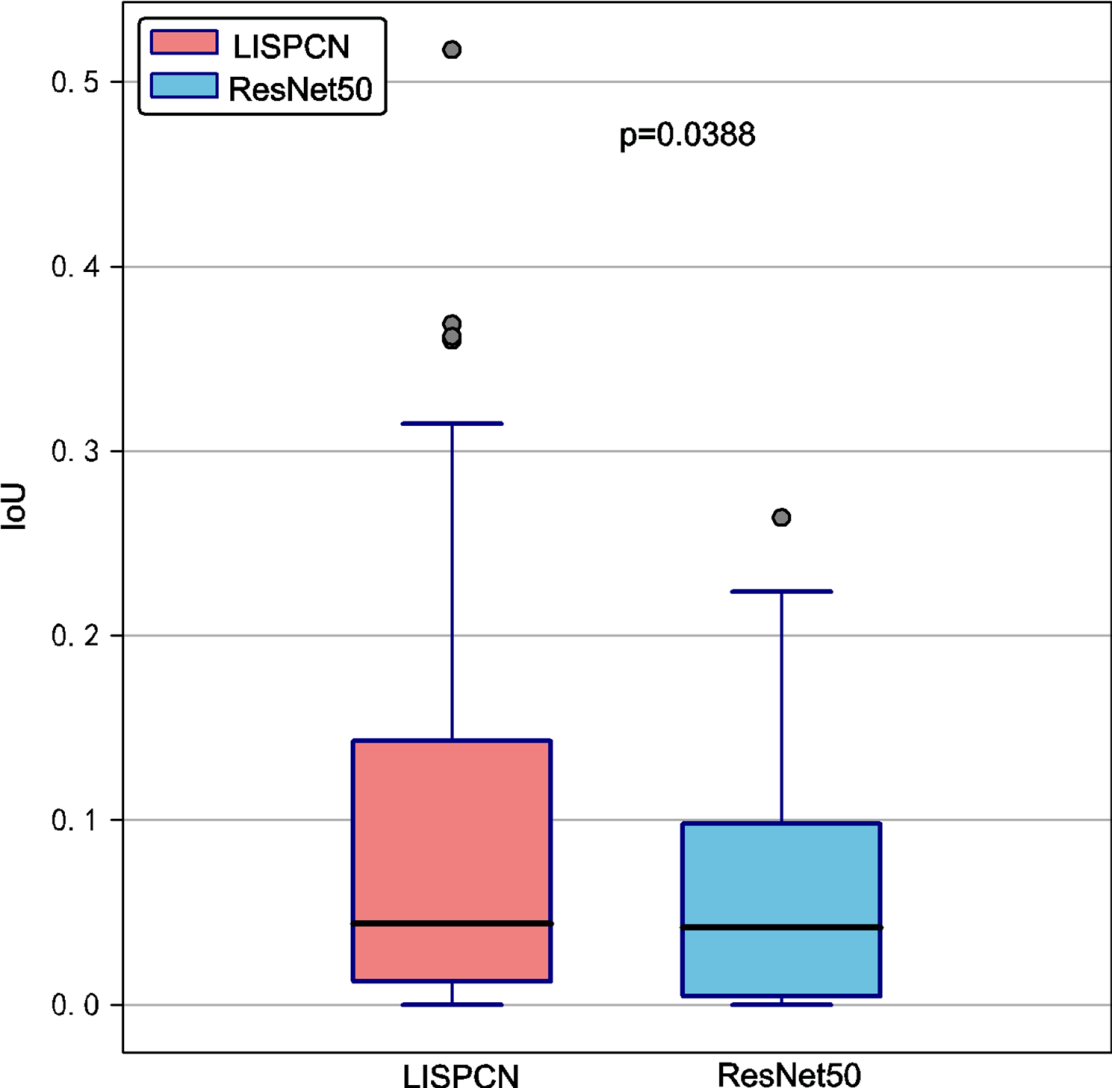
Box Plots of IoU Metrics for the LISPCN model and CNN models on the internal validation dataset

Figure 7 quantifies these differences by showing the IoU scores of the two models on the internal validation dataset. The LISPCN model consistently achieves higher IoU values, indicating its attention maps align more closely with lesion areas compared to those of the CNN model. This superior lesion-focused attention likely contributes to the enhanced therapeutic response prediction performance of the LISPCN model.

### Ablation experiments

In ablation experiments, the LISPCN method achieved the optimal prediction performance, with an AUC of 0.903 (Table 6 and Supplementary Fig. S5). Among the various groups, the AS-Dec cohort, which relies solely on a CNN for NAC response prediction, exhibited the most inferior performance across all the evaluation metrics. Moreover, the exclusion of the discriminator or generator components (AS-Dis or AS-GF) resulted in a significant decrease in prediction power compared to LISPCN (*p* = 8.32e-07 and 4.64e-06, respectively). This result underscores the potential of LISPCN in effectively learning longitudinal information and predicting final NAC responses, as opposed to the direct application of CNN on preoperative images. The ability of the AS-Pcp subgroup to predict OS was relatively superior (AUC = 0.826). This finding suggests that the introduction of perceptual loss enables comprehensive learning of information, thereby leading to more accurate predictions. The incorporation of perceptual loss not only enhances image quality but also potentially improves the accuracy of therapeutic response prediction. Thus, the results of our study elucidate the importance of various components of our proposed model for effective NAC response prediction.

**Table 6:** Experiments for the ablation study

Model	AUC (95%CI)	Accuracy	Precision	Sensitivity	Specificity	F1	*p* value
LISPCN	0.903 ± 0.021	0.813	0.835	0.848	0.763	0.842	-
AS-Dec	0.727 ± 0.073	0.689	0.764	0.522	0.847	0.620	8.32e-07
AS-Dis	0.731 ± 0.083	0.667	0.667	0.649	0.684	0.658	4.64e-06
AS-G_F_	0.785 ± 0.036	0.734	0.802	0.601	0.859	0.687	0.076
AS-Pcp	0.826 ± 0.028	0.753	0.823	0.626	0.873	0.711	0.281

## Discussion

Our study presents an approach using GANs for the simultaneous generation of early NAC images and the prediction of treatment outcomes in patients with breast cancer. The LISPCN is uniquely designed to simultaneously generate synthetic early chemotherapy images and predict neoadjuvant chemotherapy (NAC) outcomes. Utilizing a cycle GAN approach, it transforms preoperative MRI images into early-NAC images and vice versa, enhancing feature extraction and understanding of treatment-related changes. The encoder generates a latent feature map z that facilitates the joint synthesis of early-NAC MRI images and treatment response predictions. This integrated approach advances the state of the art and offers new insights into tumor response dynamics, setting our methodology apart from existing techniques. Our method improves outcome prediction accuracy and enables visual assessment of tumor morphology during early NAC, providing a valuable tool for personalized treatment planning.

In a recent study, the prediction accuracy was enhanced by combining pre- and post-NAC MR images via ensemble learning; our approach uniquely mines preoperative images to predict pCR [23]. In contrast to prior research that utilized longitudinal imaging for NAC outcome prediction, our study leverages preoperative MRI data to learn early-NAC images without the need for imaging data across treatment timelines. Additionally, another investigation [43] employed a multitask deep learning framework with Siamese subnetworks for concurrent tumor segmentation and response prediction. In contrast, our study harnessed early NAC information for simultaneous image generation and NAC response prediction. Our methodology departs from traditional longitudinal analyses by extracting temporal dynamics from preoperative images, obviating the requirement for sequential imaging.

Our study demonstrated that by mapping preoperative to early NAC images, the LISPCN model effectively learned temporal and spatial changes indicative of treatment response. The encoder module, specifically designed to capture treatment-related tumor changes, enabled the model to outperform standard CNNs which rely solely on preoperative images. Enhanced accuracy in predicting therapeutic effects was attributed to the incorporation of early NAC image-guided information, essential for capturing response-related changes. Visualization confirmed the model’s focus on relevant tumor areas, unlike the original CNN which showed decreased accuracy due to misidentification of tumor-related features.

Notably, we specifically focused on the analysis of longitudinal images taken in the early stage of the NAC regimen rather than images obtained after completion of the NAC. This strategy aligns with the findings of a previous study, which demonstrated that MRI-based response patterns halfway through the NAC can be more informative and provide more accurate predictions of therapeutic outcomes than response patterns based on images obtained after the entire NAC treatment [22]. Consequently, the information learned by the GAN model provides valuable insights into the changes and shrinkage of tumors during the early stages of NAC, which can subsequently be utilized to predict the final treatment outcomes.

The GAN-based approach predicts treatment response directly from preoperative MRI scans by generating synthetic early-NAC images, which provide oncologists with interpretable visualizations of anticipated tumor morphology changes during chemotherapy. This eliminates additional imaging sessions and enables earlier identification of non-responders than traditional post-treatment imaging methods. By capturing spatial-temporal tumor dynamics from baseline scans, the model offers pretreatment insights into expected therapeutic effects. Its dual image synthesis/response prediction functionality supports tailored decision-making, especially for predicted poor responders.

Our study has limitations that merit consideration. First, the deep GAN model was developed to synthesize early NAC images, which may reveal different positions in breast areas than preoperative images since these images were acquired at different times. Second, our models were developed by mapping preoperative images to early NAC MR images. Consequently, future investigations should consider the predictive capacity of late-NAC or even post-NAC MRI sequences for assessing pCR. Third, although we included an external validation set to demonstrate some degree of diversity, the overall sample size remains relatively modest. This limitation may affect the robustness and generalizability of our findings. Future multicenter studies ‌should utilize‌ larger, prospective datasets that include other parametric MRI or modality data to enhance the generalizability of this study. Fourth, variations in MRI scanners and imaging protocols across different cohorts may introduce inconsistencies in image characteristics, potentially impacting the model’s predictive performance. Fifth, our study is based on retrospective data, which may be subject to selection bias and limits the generalizability of our findings in clinical practice. Prospective validation studies are necessary to evaluate the model’s effectiveness in real-world clinical settings and to assess its value in guiding treatment decisions. Sixth, although our model generates synthetic early-stage NAC images, the clinical significance and interpretability of these images remain to be thoroughly examined in future research.

## Conclusion

In this study, we have developed an advanced GAN-based framework capable of simultaneously generating early-NAC MR images from preoperative scans and predicting pCR in patients with breast cancer. This approach leverages preoperative MRI to monitor morphological and functional changes during treatment, providing a comprehensive clinical assessment tool. The synthetic early-NAC images generated by our model are intended as supportive aids, not replacements for real imaging. Its clinical relevance lies in guiding personalized diagnostics and informing decisions on breast-conserving surgery and sentinel lymph node biopsy after NAC. To enhance the reliability of our diagnostic model, future studies incorporating external validation are imperative. Such investigations are crucial for verifying the model’s effectiveness across diverse patient populations and geographical regions, thereby ensuring its utility in tailoring individualized treatment plans.

## Supplementary Information

Supplementary Material 1.

## Data Availability

The ISPY-2 trial is available in TCIA on the website: [https://www.cancerimagingarchive.net/collection/ispy2/].The raw data from development and internal validation dataset cannot be publicly available but can be obtained upon official request and ethical approval by contacting the corresponding author.

## Abbreviations

SSIM: Structural similarity
PSNR: Peak signal-to-noise ratio
LJISPCN: Longitudinal image synthesis and prediction convolutional network
AUC: Area under the ROC curve
NAC: Neoadjuvant chemotherapy
GAN: Generative adversarial network
