# Supplementary material for: Joint synthesis of longitudinal MRI and enhanced prediction of neoadjuvant chemotherapy response in breast cancer: a multicohort study
Source: BMC Cancer. 2026 Mar 24;26:549. doi: 10.1186/s12885-026-15889-4 (PMC13134087; doi:10.1186/s12885-026-15889-4)
Supplement: Supplementary file 1 — Supplementary Material 1. [file 12885_2026_15889_MOESM1_ESM.docx]

**Supplementary Materials**

**Methods**

**Loss function**

Let *X* and *Y* denote the real pre- and early-NAC images, respectively, while $\hat{X}$ and $\hat{Y}$ denote the corresponding synthesized counterparts. The model’s loss function comprises three components. First, the generative loss is included, which aims to generate early-NAC images using pre-NAC images generated by generator *G*. Similarly, pre-NAC images were generated by generator *F* using early-NAC images. The cycle GAN losses are formulated as in equations 1 and 2.

$$\begin{aligned} \mathcal{L}_{GAN}\left( G,D_{Y},X,Y \right)=\mathbb{E}_{Y\sim p_{data}\left( Y \right)}\left[ \log D_{Y}\left( Y \right) \right] \\ +\mathbb{E}_{X\sim p_{data}\left( X \right)}\left[ \log\left( 1-D_{Y}\left( G\left( X \right) \right) \right) \right]\#\left( \mathrm{seq}\mathrm{equation}1 \right) \end{aligned}$$

$$\begin{aligned} \mathcal{L}_{GAN}\left( F,D_{X},Y,X \right)=\mathbb{E}_{X\sim p_{data}\left( X \right)}\left[ \log D_{X}\left( X \right) \right] \\ +\mathbb{E}_{Y\sim p_{data}\left( Y \right)}\left[ \log\left( 1-D_{X}\left( F\left( Y \right) \right) \right) \right]\#\left( seq equation 2 \right) \end{aligned}$$

where $D_{X}$ and $D_{Y}$ denote the discriminators for *X* and *Y*, respectively.

To ensure the balance of the two generators *G* and *F* for consistency between the generated image and the real images, we used the cycle consistent loss, denoted as $\mathcal{L}_{cyc}\left( G,F \right)$, formulated as follows:

$$\begin{aligned} \mathcal{L}_{cyc}\left( G,F \right)=\mathbb{E}_{X\sim p_{data}\left( X \right)}\left[ {\parallel X-F\left( G\left( X \right) \right)\parallel}_{1} \right] \\ +\mathbb{E}_{y\sim p_{data}\left( Y \right)}\left[ {\parallel Y-G\left( F\left( Y \right) \right)\parallel}_{1} \right]\#\left( seq equation 3 \right) \end{aligned}$$

To ensure that the deep features for the generated image domain and the target images have the same semantic information, we employed the perceptual loss for the generated image $\hat{Y}$ and the real image $Y$, termed $\mathcal{L}_{pcp}(G)$. Similarly, the semantic similarity between the generated preoperative image $\hat{X}$ and the real preoperative image *X*, which is termed $\mathcal{L}_{pcp}(F)$, was also evaluated. This perceptual loss is formulated by the content consistency and style consistency loss:

$\begin{aligned} \mathcal{L}_{pcp}\left( \hat{Y},Y \right)=\sum_{j} \left[ \mathcal{l}_{feat}^{\phi,j}\left( \hat{Y},Y \right)+\mathcal{l}_{style}^{\phi,j}\left( \hat{Y},Y \right) \right]\#\left( seq equation 4 \right) \end{aligned}$ where the content consistency loss and the style loss use feature maps of the networks from different layers formulated as follows:

$$\begin{aligned} \mathcal{l}_{feat}^{\phi,j}\left( \hat{Y},Y \right)=\frac{1}{C_{j}H_{j}W_{j}}{\parallel\phi_{j}\left( \hat{Y} \right)-\phi_{j}\left( Y \right)\parallel}_{2}^{2}\#\left( seq equation 5 \right) \end{aligned}$$

$$\begin{aligned} \mathcal{l}_{style}^{\phi,j}\left( \hat{Y},Y \right)={\parallel{K_{j}^{\phi}\left( \hat{Y} \right)}_{c,c^{'}}-{K_{j}^{\phi}\left( Y \right)}_{c,c^{'}}\parallel}_{F}^{2}\#\left( seq equation 6 \right) \end{aligned}$$

where $\begin{aligned} {K_{j}^{\phi}\left( Y \right)}_{c,c^{'}}=\frac{1}{C_{j}H_{j}W_{j}}\sum_{h=1}^{H_{j}} \sum_{w=1}^{W_{j}} {\phi_{j}\left( y \right)}_{h,w,c}{\phi_{j}\left( y \right)}_{h,w,c^{'}}\#\left( seq equation 7 \right) \end{aligned}$

Here, *C_j_*, *H_j_*, and *W_j_* denote the dimensions of the feature map at the *j*^th^ layer; ${K_{j}^{\phi}\left( y \right)}_{c,c^{'}}$ represent the Gram matrices of the feature maps, which are computed as the inner product between the feature map of each channel *c* and *c*'.

We constructed a semantic loss model specifically tailored to the multitask learning architecture (Supplementary Fig. S1). Consequently, the GAN loss and the classification loss were concurrently employed to train generators *G* and *F*, which are formulated through the following functions:

$$\begin{aligned} \mathcal{L}_{clf}\left( G,C_{1} \right)\mathcal{=-l}\log\left( C_{1}\left( X \right) \right)-\left( 1-\mathcal{l} \right)\log\left( 1-C_{1}\left( X \right) \right)\#\left( seq equation 8 \right) \end{aligned}$$

$$\begin{aligned} \mathcal{L}_{clf}\left( F,C_{2} \right)\mathcal{=-l}log \left( C_{2}\left( Y \right) \right)-\left( 1-\mathcal{l} \right)\log\left( 1-C_{2}\left( Y \right) \right)\#\left( seq equation 9 \right) \end{aligned}$$

where *C*_1_ and *C*_2_ are classifiers engaged in NAC for the pre-NAC and early-NAC MRI image-based predictions, respectively.

The overall function comprises the generative adversarial loss, the cycle consistency loss, the perceptual loss, and the semantic loss, as detailed below:

$$\begin{aligned} \mathcal{L}\left( G,C_{1},F,C_{2},D_{X},D_{Y} \right)=\mathcal{L}_{GAN}\left( G,D_{Y},X,Y \right) \\ +\mathcal{L}_{GAN}\left( F,D_{X},Y,X \right)+ \lambda\mathcal{L}_{cyc}\left( G,F \right) \\ +\mu\mathcal{[L}_{pcp}\left( G \right)+\mathcal{L}_{pcp}\left( F \right)] \\ +\xi\left[ \mathcal{L}_{clf}\left( {G,C}_{1} \right)+\xi\mathcal{L}_{clf}\left( {F,C}_{2} \right) \right] \#( seq equation 10) \end{aligned}$$

where $\lambda,$ $\mu$ and $\xi$ are the hyperparameters.

**Supplement Figures**

Supplementary Figure S1. Network structure. The cycle Generative Adversarial Network (cycle GAN) serves as the core architecture for synthesizing early-NAC images, featuring dual branches with paired generators and adversarial discriminators to transform images and assess their authenticity. Each generator incorporates an efficient channel attention (ECA) module to enhance discriminative capacity by focusing on crucial image regions, improving both classification and generative performance. Additionally, the model includes a generator-classifier combination that utilizes the latent feature representation z for both predictive and generative purposes, thereby optimizing model performance through balanced information sharing.

Supplementary Figure S2. The generator module jointly predicts the NAC responses and generates post-NAC images.

Supplementary Figure S3. Ablation experiments. A) AS-Dec: omitting the decoder; B) AS-Dis: removing discriminator C) AS-G: removing the generator and D) AS-pcp: elimination of perceptual loss.


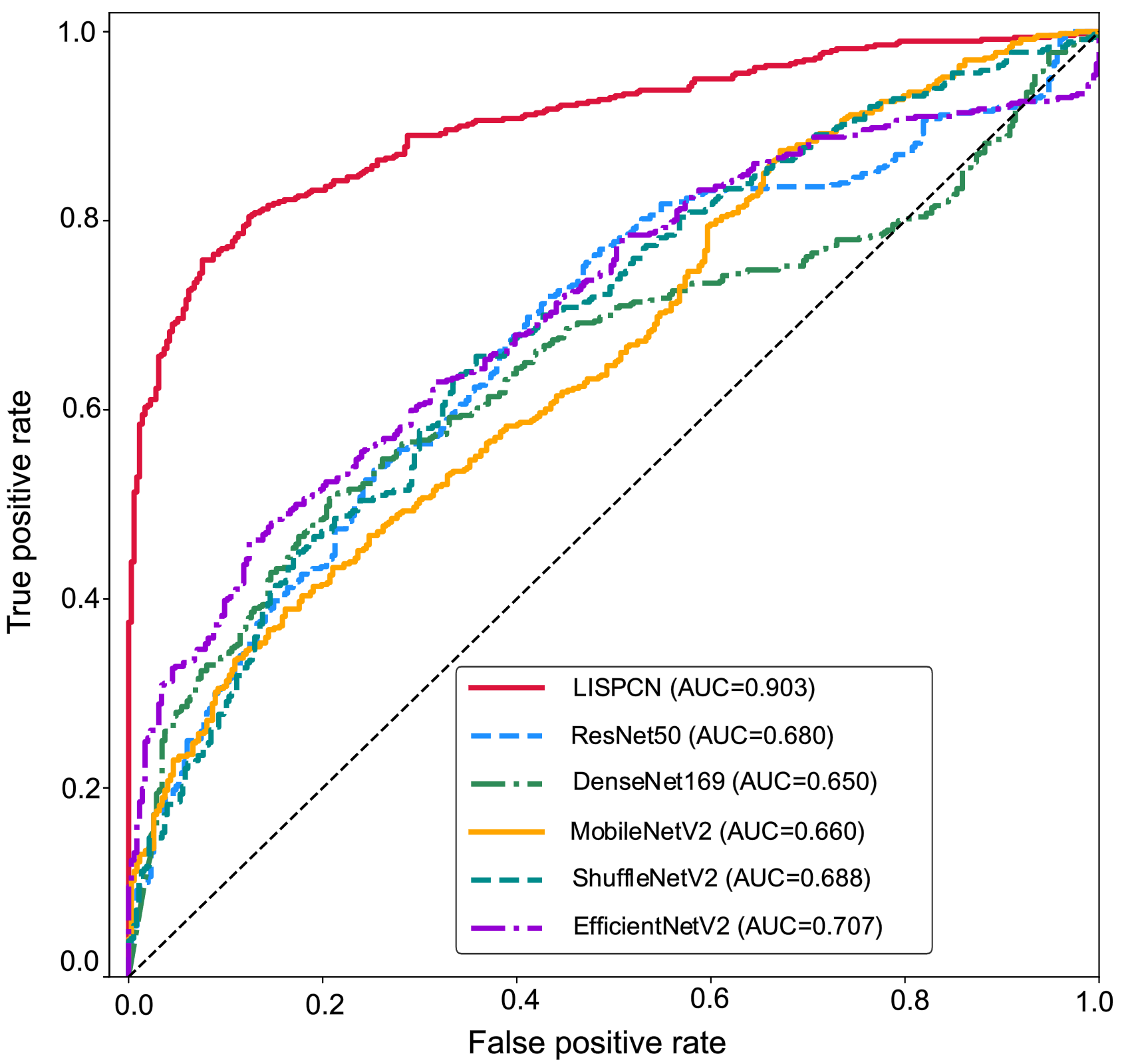


Supplementary Figure S4. Evaluation of the LISPCN model with different network backbones


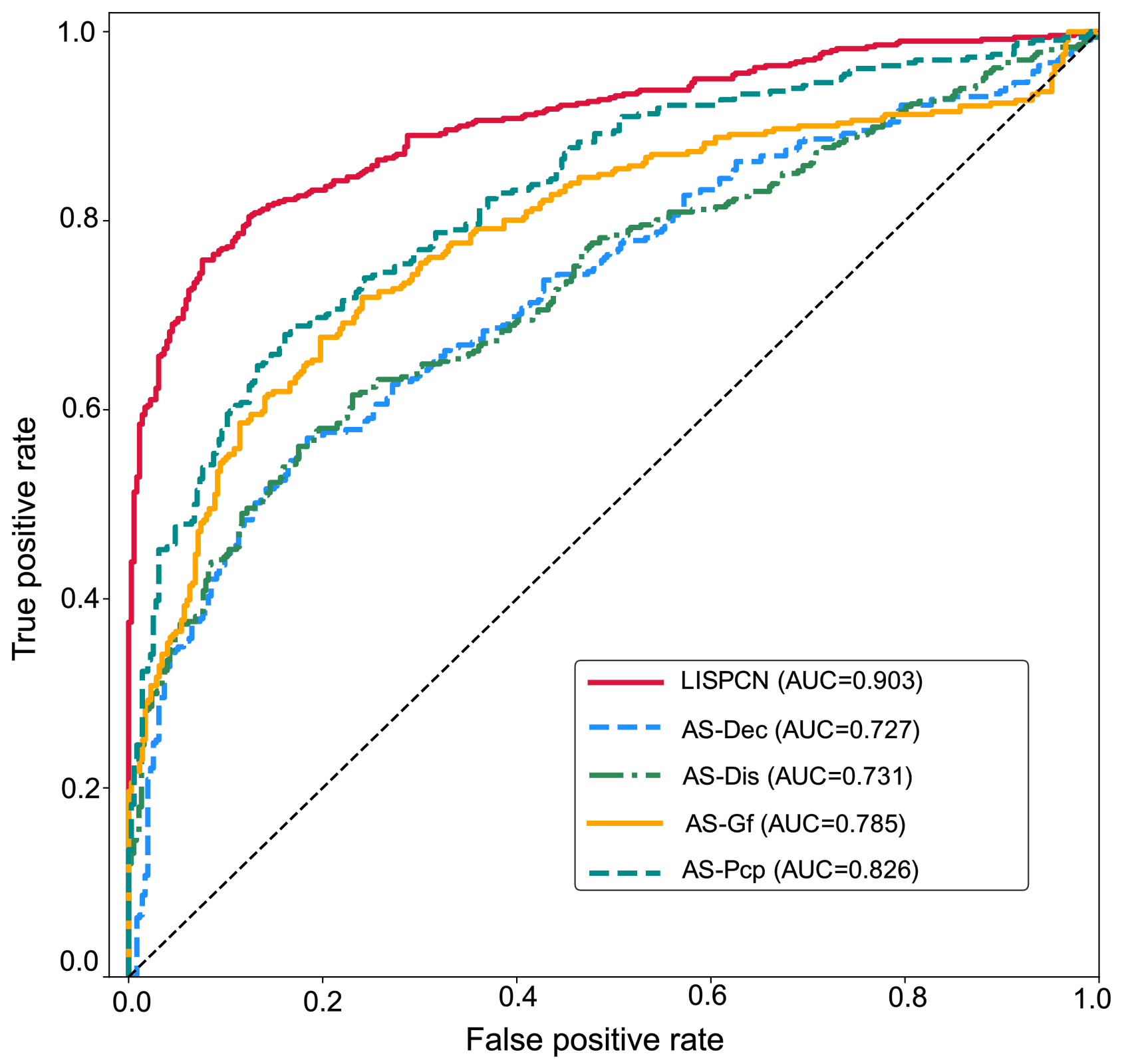


Supplementary Figure S5. ROCs for the ablation study
